# Viscous Friction between Crystalline and Amorphous Phase of Dragline Silk

**DOI:** 10.1371/journal.pone.0104832

**Published:** 2014-08-13

**Authors:** Sandeep P. Patil, Senbo Xiao, Konstantinos Gkagkas, Bernd Markert, Frauke Gräter

**Affiliations:** 1 Heidelberg Institute for Theoretical Studies, Heidelberg, Germany; 2 Technical Center, Toyota Motor Europe NV/SA, Zaventem, Belgium; 3 Institute of General Mechanics, RWTH Aachen University, Aachen, North Rhine-Westphalia, Germany; University of Leeds, United Kingdom

## Abstract

The hierarchical structure of spider dragline silk is composed of two major constituents, the amorphous phase and crystalline units, and its mechanical response has been attributed to these prime constituents. Silk mechanics, however, might also be influenced by the resistance against sliding of these two phases relative to each other under load. We here used atomistic molecular dynamics (MD) simulations to obtain friction forces for the relative sliding of the amorphous phase and crystalline units of *Araneus diadematus* spider silk. We computed the coefficient of viscosity of this interface to be in the order of 10^2^ Ns/m^2^ by extrapolating our simulation data to the viscous limit. Interestingly, this value is two orders of magnitude smaller than the coefficient of viscosity within the amorphous phase. This suggests that sliding along a planar and homogeneous surface of straight polyalanine chains is much less hindered than within entangled disordered chains. Finally, in a simple finite element model, which is based on parameters determined from MD simulations including the newly deduced coefficient of viscosity, we assessed the frictional behavior between these two components for the experimental range of relative pulling velocities. We found that a perfectly relative horizontal motion has no significant resistance against sliding, however, slightly inclined loading causes measurable resistance. Our analysis paves the way towards a finite element model of silk fibers in which crystalline units can slide, move and rearrange themselves in the fiber during loading.

## Introduction

Spider dragline or major ampullate (MA) silk is one of the toughest materials known [1], by combining not only strength and extensibility but also stiffness. It is essentially a nano-composite, in a first approximation consisting of two major components, stiff highly ordered crystalline units interspersed in a soft unstructured amorphous matrix. However, the two components are built from the same molecules, spidroin proteins, albeit with markedly different mechanical properties in the two phases.

Spidroin proteins of the mechanically robust dragline silk feature repetitive sequence motifs composed of a polyalanine (A)*_n_*, where *n* ranges from 6 to 9 amino acids [1]–[3]. These short peptides organize themselves into mechanically strong crystal blocks measuring 2–5 nm on a side [4]. These crystalline units constitute 10–25% of the fiber volume in dragline silk [1], [5]. They are followed by a glycine-rich motifs that form the amorphous phase, which are composed of (GGX)*_n_* and (GPGXX)*_n_*, with *n* is in the range from 20 to 30 amino acids [1]. The amorphous region is predominantly disordered [2], [6], [7] and the peptide sequences are oriented along the fiber axis during stretching experiments [1],[8],[9]. Thus, dragline silk is a semicrystalline material with crystals of a well-defined nanometer size reminiscent of a nanocomposite, which is imprinted by the protein sequence.

The high stiffness and yield strength of silk fibers have been attributed to the *β*-sheet crystals, whereas the high extensibility is thought to arise from the amorphous glycine-rich domains [10]. Molecular Dynamics (MD) simulations have increasingly helped to gain further insight into the force-bearing structures and interactions within the crystal [11]–[13] and the amorphous phase under mechanical load [14]. The crystalline component of silk fibers behaves like an elasto-plastic material, which undergoes non-reversible rupture in response to applied forces [15]. The amorphous phase is softer and features a rate-dependent behavior, i. e., it is a viscoelastic material [14].

However, to our knowledge, the mechanical resistance, or friction, at the crystalline-amorphous protein-protein interface within the fiber has not yet been characterized to date. Depending on the extent of interfacial friction, crystals are able to slide, and thereby redistribute within a silk fiber under mechanical load. Such a mechanism has not been considered in the existing structure-based fiber models of dragline silk [10], [13], [16]–[19].

Molecular friction has been systematically assessed for proteins at inorganic (hydrophobic or hydrophilic) surfaces using both single molecule force spectroscopy and MD simulations [20]–[22]. We here focus on the friction between the amorphous and crystalline domains of dragline silk fibers. We assessed friction forces at the interface by MD simulations, and from there deduced a coefficient of viscosity at the viscous limit, analogously to our previous determination of the coefficient of viscosity within the amorphous phase [14]. We employed the coefficient of viscosity in proof-of-principle finite element models. Our quantitative analysis of the friction between them presents an important step towards developing a bottom-up visco-elastoplastic model for dragline silk fibers.

## Materials and Methods

### Molecular Dynamics Simulations

We constructed a composite model of crystalline units and the amorphous phase of spider silk from the MA gland of *Araneus diadematus* [1]. We built crystalline units composed of the repeat units found to be present in *Araneus diadematus* spider silk fibers, AAAAAAAA. We arranged five layers of *β*-sheets, each consisting of five *β*-strands of the respective sequence, such that the model exhibits optimal hydrogen bonding, as previously described [12].

**Figure 1:**
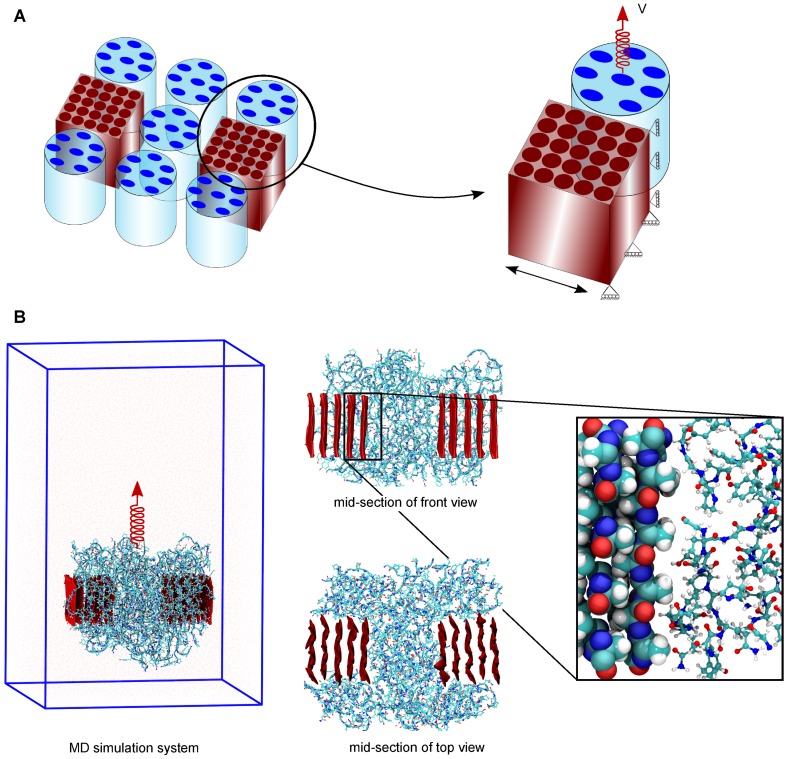
Setup of an FPMD simulation for assessing molecular friction between seven bundles of the amorphous phase and two crystalline units. (A) Schematic representation of the model before equilibration (left). The two crystalline units (red) are 3 nm apart, and seven bundles of the amorphous phase (blue) are placed around it. The loading and boundary conditions of the model are indicated (right). A harmonic spring that moves with constant velocity *V* was connected to the termini of the seven bundles. The crystalline units were position-restrained in pulling and in one lateral direction. (B) The MD simulation system (left) with a front and top view of mid-sections (middle), and an enlarged view of interactions between the crystalline and amorphous component (right).

Next, we constructed the amorphous phase from a representative 24-residue sequence (GPGGYGPGSQGPSGPGGYGPGGPG, where G, P, Y, S, Q are glycine, proline, tyrosine, serine, and glutamine, respectively) of *Araneus diadematus* MA silk [14]. We constructed our friction model such that two crystalline units were 3 nm apart, and seven bundles of the amorphous, each containing eight fully stretched peptides, were positioned between and around these two units (Figure 1A). This model, in total consisting of 50 crystalline and 56 amorphous peptides, was constructed using the software Visual Molecular Dynamics (VMD) [23]. For subsequent MD simulations, we used the GROMACS 4.5.3 package [24], and the OPLS-AA force field [25] for the protein. Simulation boxes of 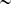
12.0×18.6×12.0 nm^3^ were used.

The crystalline units and the amorphous phase models were subsequently solvated in TIP4P water [26]. We note that semi-ordered regions, supposedly formed by (GA)*_n_* repeats, were not part of our simulation system. Here, we combined the two extremes only, fully ordered and fully disordered phases. The solvent included Na and Cl ions with a concentration of 0.1 mol/liter, resulting in a system size of 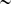
0.31 million atoms. Periodic boundary conditions were employed to remove artificial boundary effects. We chose a cutoff of 1.0 nm for non-bonded interactions, and the Particle Mesh Ewald (PME) method [27] to account for long-range electrostatic interactions. To increase the simulation time step, we used LINCS [28] to constrain all bond vibrations. A time step of 0.002 ps was used. Simulations were performed in the NPT (isothermal-isobaric) ensemble with a temperature of 300 K and a pressure of 1 bar. We used Nosé-Hoover [29], [30] temperature coupling with a coupling time constant 0.1 ps, and Parrinello-Rahman [31], [32] pressure coupling with a coupling time constant of 1 ps. The simulation systems were relaxed by energy minimization. We then performed 500 ps position-restrained simulations to equilibrate the solvent, subjecting each protein atom to a harmonic potential with a force constant of 1660 pN/nm. Finally, all models were fully equilibrated for 200 ns allowing the silk peptides to adopt relaxed conformations and to partially entangle within the amorphous phase as well as with the crystalline units. The resulting equilibrated simulation systems served as starting points for force-probe Molecular Dynamics (FPMD) simulations [33].

In the FPMD simulations, seven bundles of the amorphous phase were pulled in the upward direction, as schematically shown in Figure 1A, B. Forces were applied by attaching one-dimensional harmonic springs with a force constant of 830 pN/nm acting at the center of mass of the terminal residues of the each bundle of the amorphous phase. The goal of this study was to compute friction between the amorphous phase and crystalline units as they slide relative to each other. Therefore, the crystalline units were position-restrained along the pulling direction as well as in one lateral direction (as shown in Figure 1A), subjecting each protein atom in this part to a harmonic potential with a force constant of 1660 pN/nm. The springs were moved with constant velocities ranging between 0.02 and 20 m/s. There was no external force exerted perpendicular to the pulling direction on the crystalline units as well as the amorphous phase. The FPMD simulations were stopped after the amorphous phase detached from the crystalline units.

To obtain a shear stress, which then can be converted into a coefficient of viscosity, we calculated the contact area between the amorphous phase and crystalline units which gives rise to the resistance area against sliding. Microscopic contact areas between a polymer surface and a crystalline surface as well as between polymer surfaces have been previously calculated on a molecular scale to determine shear stresses [34], [35]. Here, we used the solvent accessible surface area (SASA) as the contact area between the amorphous phase and crystalline units. We subtracted the SASA of the whole protein (the amorphous phase bundles and crystalline units) from the sum of the areas of only the amorphous phase bundles and crystalline units, when considered in isolation, which then, after division by two, gives the interface or contact area. We used a solvent probe of radius 0.14 nm (recommended probe radius for water) for this purpose [24].

### Finite Element Model

For the finite element calculations of the friction model, the commercial solver LS-DYNA (version: ls971s R5.1.1) [36] was used together with the Pre/Post tool LS-Pre-Post [37]. A rectangular cube of the crystalline unit and a rectangular plate of the amorphous phase were modeled by using 8 node hexahedral (brick8) elements. In our previous work, we studied the crystalline component [15] as well as the amorphous phase of *Araneus diadematus* dragline spider silk [14]. In that study, we concluded that the crystal component behaves like an elastoplastic material, which was described with the *MAT_003 material model (suited to model isotropic and kinematic hardening plasticity) [36]. The amorphous phase has a rate-dependent behavior, which accordingly was described with a viscoelastic material model, *MAT VISCOELASTIC (*MAT_006) [36].

In this work, we modeled the contact between the amorphous phase and crystalline unit as *CONTACT SURFACE TO SURFACE. Generally in LS-DYNA modeling, the contact between two bodies is defined by an interface made up of slave and master sides. This is a two-way treatment of a contact, which means that both the master and slave surfaces of the contact are checked for penetration during the simulations. In any explicit-integration scheme, it is imperative that for proper load transfer between the two bodies in contact the slave side mesh is finer than the master side mesh. For this simulation, the amorphous phase is taken as the slave side, while the crystalline unit is defined as the master side. Friction in LS-DYNA is calculated by a Coulomb friction formulation [36].

The segment-based penalty method for a contact was used in this work. The interface surfaces were modeled by two dimensional (2D) shell elements with the null material (*MAT_009). It is advantageous to model contact surfaces via shell elements which are not part of the structure, but this requires to define areas of contact between the bodies. The null material behaves in a fluid-like manner, and we assigned as input parameter the dynamic viscosity coefficient which was obtained from the above MD study. In our friction model, the amorphous component plate was restrained in an upward direction but kept free in the other two direction, and load was applied to the cube of the crystalline unit.

## Results and Discussion

To assess the frictional forces between the amorphous and crystalline phase of spider silk at atomistic scale, we here used atomistic FPMD simulations. We closely followed the protocol that we previously employed to assess frictional forces within the amorphous phase [14]. The simulation setup is depicted in Figure 1A and B, showing a schematic representation of the simulation system with boundary conditions and the actual simulation system, respectively. In our simulations, we used two crystalline units of 5×5 strands and seven bundles of the amorphous phase of *Araneus diadematus* spider silk (for details on the model setup and boundary conditions see Materials and Methods).

**Figure 2:**
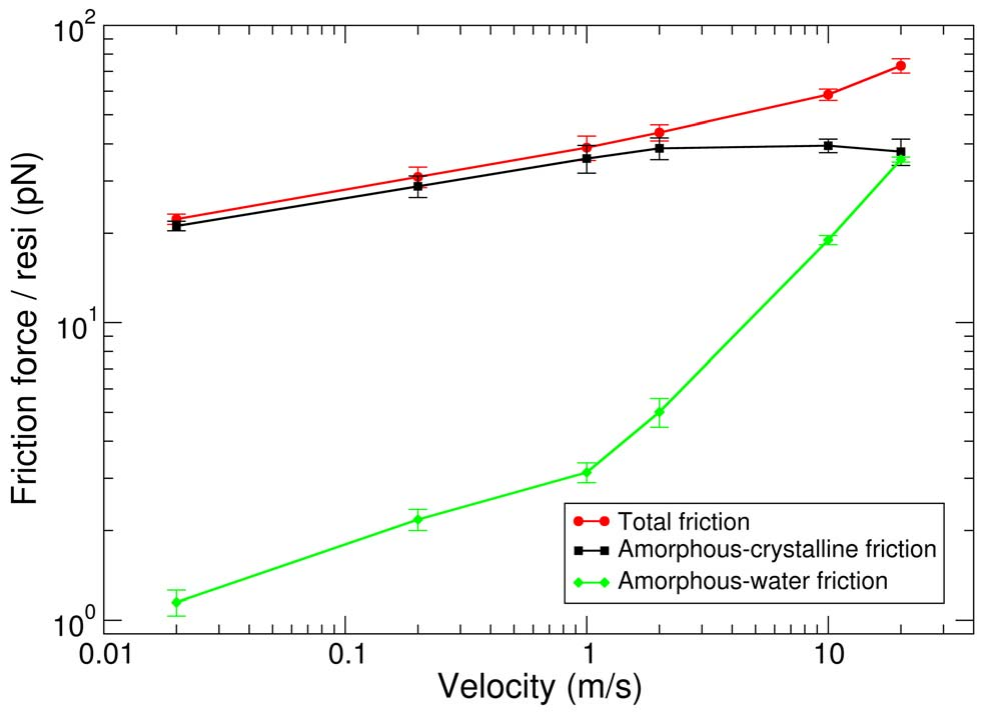
Friction force per residue (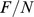) as a function of pulling velocity (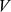) when pulling the amorphous phase along the crystalline units. Both amorphous-crystalline and amorphous-water friction contribute to the total friction.

A harmonic spring was connected to the termini of the seven bundles of the amorphous phase, and moved at constant velocity, while the other termini of the bundles were kept free to move. By pulling out seven bundles of the amorphous phase, away from the crystalline units they surround, we could measure the friction force upon sliding the amorphous phase relative to the crystalline units. We obtained peak frictional forces for both amorphous-crystalline and amorphous-solvent friction for different pulling velocities. We next separated the frictional forces of the amorphous phase with crystalline units from frictional forces with water. To this end, we compared the peak forces obtained for the sliding of bundles of the amorphous phase to the peak force required to pull these bundles with the same pulling velocity through water, as observed in additional FPMD simulations with all bundles pulled in the same direction without crystalline units. Figure 2 shows the frictional force per residue for dissociating the bundles from crystalline units (red), for dragging them through water (green), and their difference, i. e., the amorphous-crystalline friction (black). Data was obtained at different pulling velocities, and averages and standard errors over four independent FPMD simulations are given. Note that the friction force corresponds to an effective mean force, i. e., we assume that force is on an average equally shared by all contacting residues between the two phases.

For low velocities (<2 m/s), total friction forces and amorphous-crystalline friction forces are of similar magnitude, i. e., water gives rise to an only minimal resistance against the sliding of the amorphous phase. For velocities beyond 2 m/s, amorphous-water friction substantially contributes to the total frictional force. We note that we did not observe water molecules inbetween the amorphous phase and crystalline units, so that friction with water is effectively restricted to the outer surface of the amorphous phase in our simulation system, i. e., amorphous-crystalline friction is dry (Figure S1 in File S1). Figure 2 shows that the water friction force grows nearly linearly with applied velocity. A straight line would follow the linear viscous law, 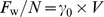 [21], where *F*_w_ is the water friction force, *N* is the number of residues are in contact, and *V* the applied constant velocity. From the simulations, the per-residue friction coefficient, 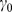, with water is 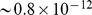 Ns/m, which is very close to the experimental value of bulk water of 1×10^−12^ Ns/m [38], , and the same as determined previously for a single amorphous bundle [14].

### Viscous Friction Coefficient

In the experiments, such as force spectroscopy experiments or when biological molecular motors are active, the applied external force causes molecular motions in the *µ*m/s range. Therefore, time scales of experiments typically fall into the viscous linear response regime, where friction forces are proportional to velocities. Thus, to extract the viscous friction coefficient from the sliding of the amorphous phase relative to the crystalline units in our simulations, we have to extrapolate our data to the viscous regime. In the viscous regime, the coefficient of viscosity does not depend on the shear stress. For an extrapolation as robust as possible, we tried to push the limit towards longer time scales as far as we could. In our simulations, we were only able to reach 0.02 m/s as lowest pulling velocity, which, however, is still distant from the viscous regime.

In this work, we used a stochastic model which describes the full velocity dependence of the friction force per residue [21]. In our previous work, the earlier proposed stochastic model [21], [22] was modified to extract the viscous friction coefficient from the sliding of silk peptide chains [14]. Here, analogously, the friction coefficient per residue can be calculated according to
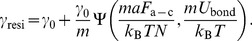
(1)


Here, *a* is a lattice constant, *m*, the cooperativity of bonds, *U*_bond_ the bond strength, and 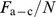 denotes the frictional force between the amorphous and crystalline phase per residue. In the Fokker-Planck equation, a bond refers to an adhesive bond, in our case between the amorphous and crystalline phase. Note that we assume the friction force 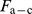 to be equally distributed on all *N* residues. The stochastic model is used to fit the simulation data set by varying the bond cooperativity, the strength of bonds, or the lattice constant. In this way, we could extract the coefficient of viscosity 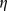 or the viscous friction parameter between the amorphous phase and crystalline units of spider dragline silk from our MD simulations. We also extracted a friction coefficient per residue, as shown in Figure S2 in File S1.

**Figure 3:**
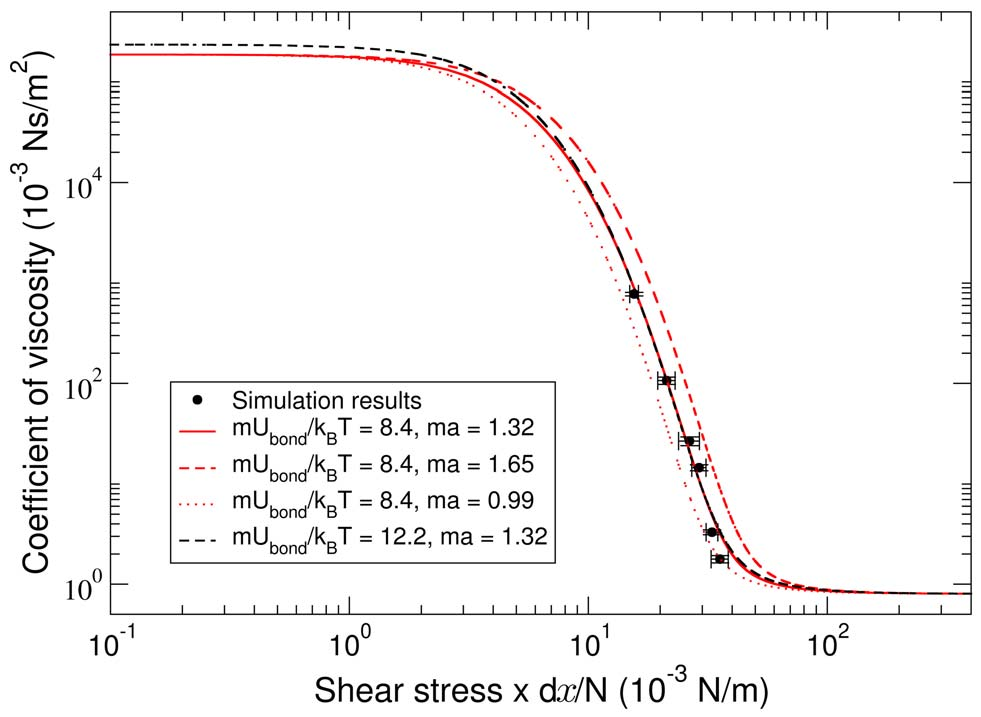
Simulated coefficient of viscosity per residue as a function of shear stress 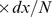. Red and black lines present fits of the stochastic model to the simulation data with varying *ma* and 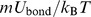, respectively. The solid red line shows the best fit to the data.

The coefficient of viscosity 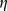 is defined by Newton's law of shear viscosity, with 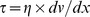, where 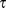 is the shear stress, and 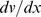 is the shear velocity or velocity gradient. Figure 3 shows the coefficient of viscosity per residue, 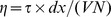 from the solution of the Fokker-Planck equation, as a function of 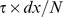. When fixing the strength of individual residue bonds to the value 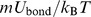 = 8.4, and treating the periodicity *a* as fitting parameter, which controls the lateral position of the scaling function (red lines in Figure 3), we obtained a value 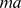 of 1.32±0.33, which covers the range of the simulation data. Fixing the parameter *ma* to 1.32 and varying the strength of individual residue bonds (red solid and black line in Figure 3) yields a strength of 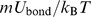 = 10.3±1.9. Fits with 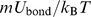 of 8.4 and *ma* of 1.32 are representing the data best (solid red line).

From our simulation results, the coefficient of viscosity or dynamic viscosity per residue with water at high velocity of 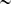
0.8×10^−3^ Ns/m^2^, which is same as that of our previous work [14], and also close to the experimental value of the dynamic viscosity of water 1×10^−3^ Ns/m^2^ [40], [41]. Note that the crystals feature two distinct surfaces, one hydrophobic surface type with exposed alanine sidechains parallel to the *β*-sheet plane in the crystals, and another hydrophilic type with backbone hydrogen bond acceptors and donors. These two types might feature different levels of friction, but we here obtained an effective coefficient of viscosity representing the average friction on the both surface types.

We obtained a coefficient of viscosity or the viscous friction parameter between the amorphous phase and crystalline units of spider dragline silk of 2±0.2×10^2^ Ns/m^2^. The obtained coefficient of viscosity is two orders of magnitude lower than that within the amorphous phase, which is 1±0.5×10^4^ Ns/m^2^ [14]. We note that the actual uncertainty in the coefficient of viscosity is larger than what the given error suggests. The reason is that the data does not fall into the turnover regime into the viscous limit, as we do not achieve sufficiently long time scales in our MD simulations. However, the fitting was robust with respect to variations of 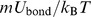, and the best fitting values fall into the previously obtained range of values [14],[21],[22]. Interestingly, the bond strength we obtain here is lower than the one we obtained within silk peptide bundles, which reflects the lower hydrogen bond density at the partially hydrophobic crystal surfaces. We emphasize that the extrapolation serves as an estimate largely for the order of magnitude of the coefficients derived for subsequent upscale finite element simulations.

### FEM to Viscous Friction

We next determined the friction between the crystalline and amorphous blocks by finite element modeling, using the coefficient of viscosity determined from MD simulations as described above. The mechanical properties of the elastoplastic crystalline [15] and the viscoelastic amorphous phase [14] were directly adopted for these FEM simulations.

**Figure 4:**
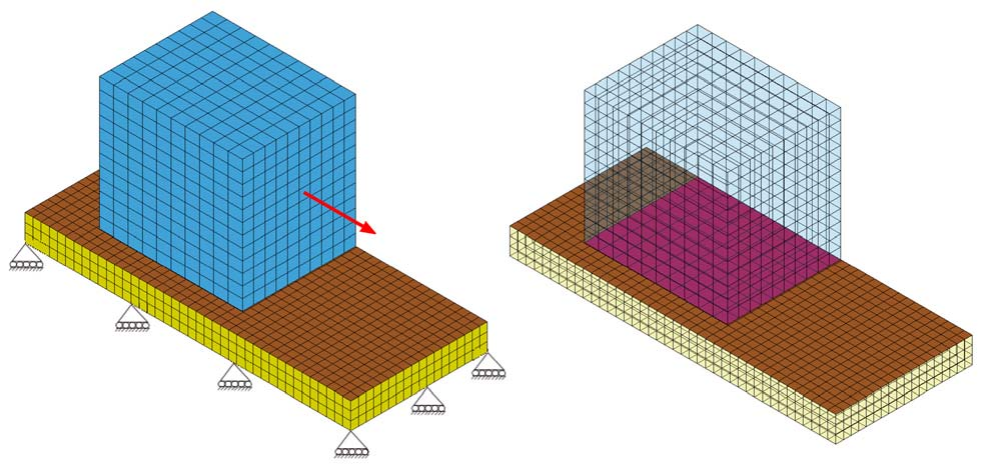
Finite element modeling of interfacial friction. Schematic picture of the finite element model with boundary conditions (left). The model includes the crystalline unit (blue), the amorphous phase (yellow), and contact surfaces (right). The master segment was assigned to the crystalline unit surface (magenta), and the slave segment to the amorphous phase surface (brown).

To describe the friction in this model, we included a lubrication film of 2D shell elements of the null material between the amorphous phase and the crystalline unit. The lubrication film was defined with contact surfaces as shown in Figure 4 (right). Load was applied to the crystalline cube and the amorphous plate was fixed at the bottom as shown in Figure 4 (left). The load was transferred from the cube to the amorphous plate through the viscous lubrication film. We assigned a coefficient of viscosity to the contact surfaces, as obtained from MD simulations (see above). The viscous layer gives rise to a sliding friction between them in a rate-dependent manner.

**Figure 5:**
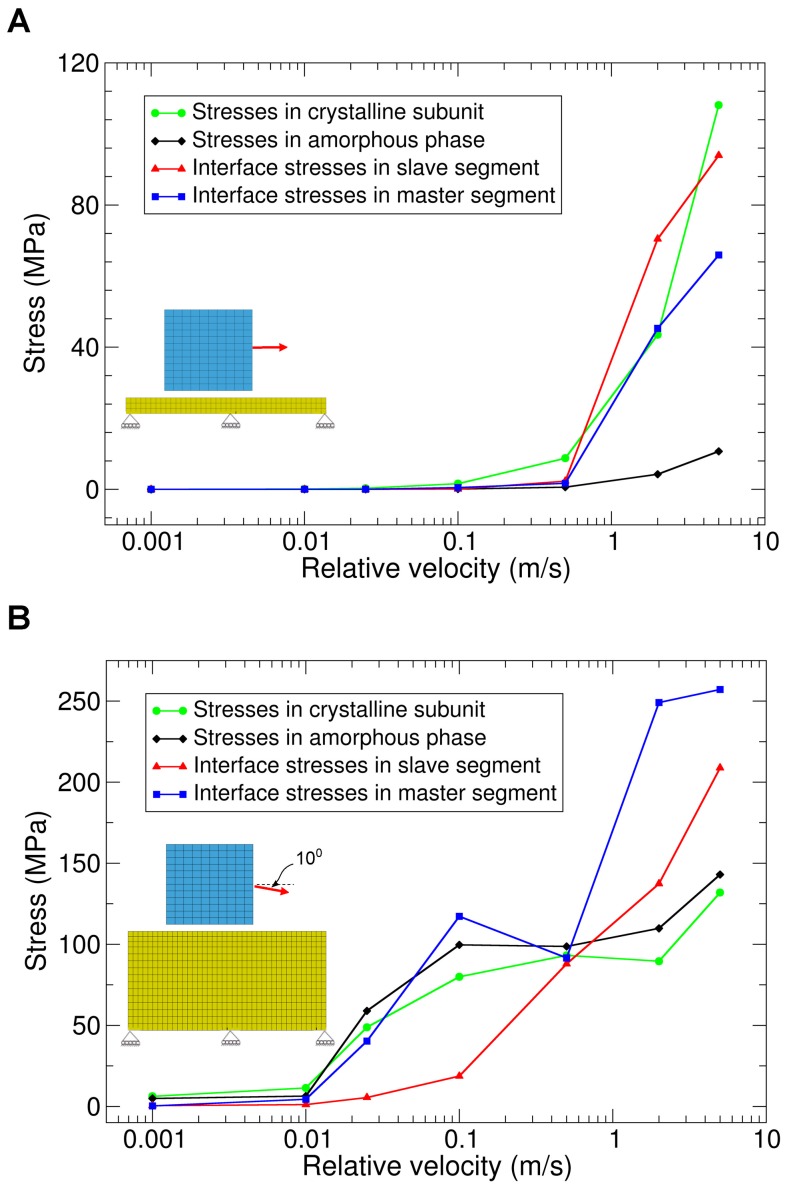
Stresses in the crystalline and amorphous components as well as the interface segments as a function of their relative velocity. (A) The crystalline cube was pulled horizontally along the amorphous rectangular plate of 0.5 nm thickness. (B) The crystalline cube was pulled with a 10 degree angle with respect to horizontal plane along the amorphous component of 2.85 nm thickness.

Figure 5 shows stress as a function of relative velocity for the friction model. Here, we studied two load cases as idealized cases of the complex situation in tensed silk fibers. In the first loading case, the crystalline cube was pulled horizontally along the amorphous plate, corresponding to a force parallel to a silk fiber axis. For fast relative velocities (>1 m/s), stresses in the crystalline component as well as in the interface were of high magnitude, and increased with increasing relative velocities. For low relative velocities (<1 m/s), stresses in both components and in the interface were not significant (nearly zero) as shown in Figure 5A. However, no significant stresses occurred in the amorphous phase for all relative velocities. In the second loading case, the crystalline cube was pulled along a direction forming a 10 degree angle with the horizontal amorphous plate (Figure 5B). This slight loading inclination, stresses upon relative sliding of the two phases were significant even for velocities smaller than 1 m/s.

Taken together, our FEM simulations predict that in the situation of perfect relative horizontal motion, there is no significant resistance against sliding at low to medium velocities. However, slightly inclined loading may cause substantial resistance to the sliding relative to each other, and resistance increases with increasing relative velocities.

## Conclusions

Here, we have quantified the viscous friction between the amorphous phase and the crystalline unit of *Araneus diadematus* silk using MD simulations. The coefficient of viscosity for this interface is in the order of 10^2^ Ns/m^2^. Remarkably, this value is two orders of magnitude smaller than the coefficient of viscosity we previously obtained for the relative sliding of peptides within the amorphous phase [14]. Thus, disordered peptides of the amorphous matrix slide along the surface of crystalline units with much less hindrance as opposed to sliding within other entangled disordered chains. This large difference of the two proteineous components can be due to both the difference in their sequence and structure. The crystalline unit comprises only alanine residues, i. e., sidechains of relative small and homogeneous size, thereby minimizing the molecular ruggedness of the crystalline surface. Secondly, the surfaces of crystalline units are flat also due to the straight *β*-strand configuration therein, as opposed to the entanglement of proteins within the disordered amorphous matrix.

Our finite element model allowed to quantify the frictional behavior between these two components as it might occur within the context of silk fibers for the experimental range of pulling velocities. We concluded that a perfectly relative horizontal motion has no significant resistance against sliding. However, a slightly inclined loading may cause a high resistance to sliding. Our results within this simple and very reduced model suggest that crystalline units can slide, move and rearrange themselves in the fiber during loading, a scenario with potential impact for silk mechanics.

On the basis of MD simulations and deduced parameters presented herein and previously [13]–[15], a refined finite element model of full silk fibers can be established, in which crystalline units are connected to the amorphous phase along the fiber axis direction, while no connections but frictional forces are defined between them in the direction perpendicular to the fiber axis. Such a bottom-up computational model eventually allows to test the role of relative sliding and frictional forces between the ordered and disordered phases for the outstanding toughness of silk fibers.

## Supporting Information

File S1 Solvent 2D number-density maps of simulation system and friction coefficient per residue using the solution of the Fokker-Planck equation.
(PDF)
