# Supplementary material for: Viscous Friction between Crystalline and Amorphous Phase of Dragline Silk
Source: PLoS One. 2014 Aug 13;9(8):e104832. doi: 10.1371/journal.pone.0104832 (PMC4132047; doi:10.1371/journal.pone.0104832)

Supporting Information

Viscous Friction between Crystalline and Amorphous Phase of Dragline Silk

Sandeep P. Patil, Senbo Xiao, Konstantinos Gkagkas, Bernd Markert, Frauke Gräter

Figure S1. Solvent 2D number-density maps. Solvent number density along the z direction and averaged over the whole protein (the amorphous bundles and crystalline units) width (left) and density along the y direction and averaged over the whole protein length (right). The amorphous-crystalline friction is a result of the adhesive bonding between the crystalline and amorphous phase, and an only negligible amount of water is located within the peptide bundle, as shown by the solvent 2D number-density maps. Thus, the amorphous-crystalline friction is dry.

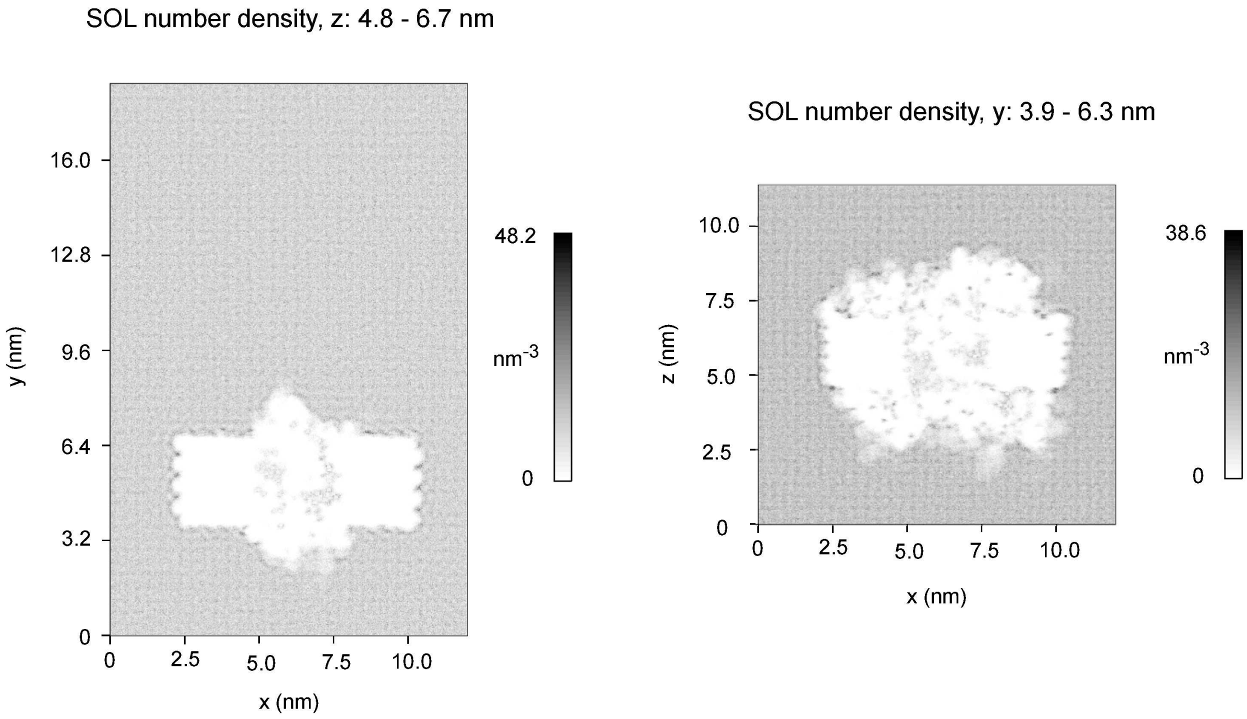

Figure S2. Comparison of the simulation data set with the solution of the Fokker-Planck equation. Friction coefficient between the amorphous phase and crystalline units per residue as a function of friction force per residue. The stochastic model is used to fit the simulation data set (blue) by varying ma and $mU_{\rm bond}/k_{\rm B}T$. When fixing the strength of individual residue bonds to the value $mU_{\rm bond}/k_{\rm B}T = 8.0$, and treating the periodicity a as fitting parameter, which controls the lateral position of the scaling function (red lines), we obtained a value ma of 2.10 ± 0.85, which covers the range of the simulation data. Fixing the parameter ma to 2.10 and varying the strength of individual residue bonds (red solid and black lines) yields a strength of $mU_{\rm bond}/k_{\rm B}T = 8.0 \pm 1.8$. Using these fit parameters, for high velocities, the friction coefficient obtained from simulations is $\sim 0.8 \times 10^{-12}$ Ns/m. The extrapolation to low velocities gives a friction coefficient per residue for the friction interface between the amorphous phase and crystalline units of spider silk of $1.8 \pm 0.6 \times 10^{-7}$ Ns/m.

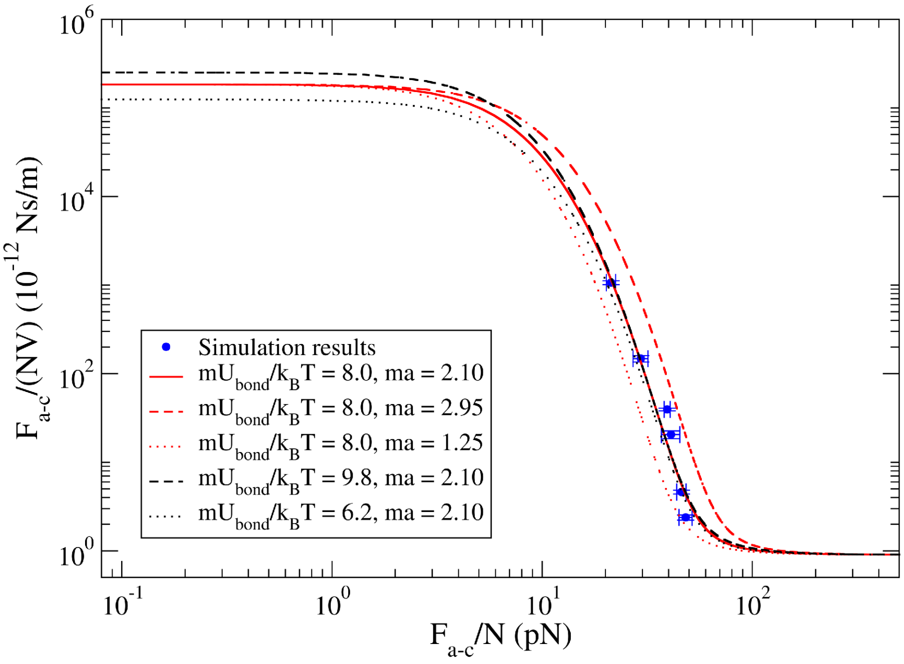

Supplement: File S1 — Solvent 2D number-density maps of simulation system and friction coefficient per residue using the solution of the Fokker-Planck equation. (PDF) [file pone.0104832.s001.pdf]
